# Personalized Bilateral Upper Limb Essential Tremor Therapy with Botulinum Toxin Using Kinematics

**DOI:** 10.3390/toxins11020125

**Published:** 2019-02-19

**Authors:** Olivia Samotus, Jack Lee, Mandar Jog

**Affiliations:** 1 Department of Clinical Neurological Sciences, London Health Sciences Centre—Lawson Health Research Institute, 339 Windermere Road, A10-026, London, ON N6A 5A5, Canada; osamotus@uwo.ca (O.S.); jack.lee@lhsc.on.ca (J.L.); 2 Schulich School of Medicine and Dentistry, University of Western, 1151 Richmond Street, London, ON N6A 3K7, Canada

**Keywords:** movement disorders, essential tremor, functional disability, Botulinum toxin, kinematic tremor analysis, computer-assisted, biomechanics

## Abstract

Variability of multi-joint essential tremor (ET) between patients and within the two upper limbs makes a visual assessment for the determination of botulinum toxin type A (BoNT-A) injections challenging. Kinematic tremor analysis guidance has succeeded in overcoming this challenge by making effective long-term unilateral BoNT-A injections for disabling ET. In this open-label study, 31 ET participants received three bilateral arm BoNT-A injection cycles over 30 weeks with follow-ups six-weeks post-treatment. Whole-arm kinematic assessment of tremor using a customized, automated algorithm provided muscle selection and dosing per muscle without clinician’s assessment. Efficacy endpoints included Fahn-Tolosa-Marin tremor scale, quality of life (QoL) questionnaire, and maximum grip strength. BoNT-A reduced tremor amplitude by 47.7% in both the arms at week-6 (*p* < 0.005) that persisted from weeks 18–30. QoL was improved by 26.5% (*p* < 0.005) over the treatment period. Functional interference due to tremor was reduced by 30% (*p* < 0.005) from weeks 6–30. Maximum grip strength was reduced at week 6 (*p* = 0.001) but was not functionally impaired for the participants. Effective bilateral ET therapy by personalized BoNT-A injections could be achieved using computer-assisted tremor analysis. By removing variability inherent within the clinical assessments, this standardized tremor analysis method enabled patients to have improved bimanual upper limb functionality after the first treatment.

## 1. Introduction

During the first year of diagnosis, 30% of essential tremor (ET) patients are non-responders to oral medication (e.g., primidone, propranolol), while in the following year, an additional 30% of patients discontinue their treatment due to a lack of satisfaction from poor functional benefit and an increase in adverse effects [1,2]. Upper limb tremor severity can be further worsened causing disability to bimanual arm function, during activities of daily living, for work-related tasks, and hence impairs quality of life (QoL). In our previously reported open-label trials, ET patients were treated unilaterally in their most disabling tremulous arm, as perceived by the patient, with personalized botulinum toxin type A (BoNT-A) injections [3,4]. In serial treatments (every 16 weeks), significant alleviation of tremor and improved arm functionality was observed six weeks following the first treatment, continued up to 96 weeks [3,4], and is currently ongoing. Thus, bilateral upper limb treatment of ET may reduce improvement time and further improve arm functionality and QoL [5]. 

The importance of using a personalized BoNT-A approach for treating tremor has now been supported by recent clinical studies as the best method to achieve tremor reduction with minimal hand weakness [6,7]. In our studies, personalized therapy of BoNT-A was achieved using computer-assisted, kinematic tremor analysis at the wrist, elbow, and shoulder aiding the clinical judgment of injection parameters (the selection of arm muscles contributing to tremor and the appropriate dose per muscle) [4]. BoNT-A injection parameters can now be fully determined by this computer-assisted tremor analysis. 

This is the first study to report the efficacy and safety of bilateral arm BoNT-A (incobotulinumtoxinA) injections for tremor using objective measurements and clinical outcomes for activities of daily living, quality of life, and maximal grip strength. In this 30-week clinical trial, ET patients with a functionally debilitating tremor in both arms were injected with BoNT-A, where injection parameters were entirely determined by computer-assisted tremor analysis in wrist, elbow, and shoulder muscles per arm.

## 2. Results

### 2.1. Participant Demographics

Of the 35 ET participants consented, 31 individuals met all the inclusion criteria. The participants’ demographics and baseline clinical scores are presented (Table 1). Four participants were withdrawn on the first study visit due to: inability to use needle electromyography (EMG) injection due to obesity and needle length, a recent change in health unknown to investigator prior to consenting, and tremor in both arms but only one arm with debilitating tremor. Thirty-nine percent of participants (12/31) continued their oral medication, such as propranolol (mean daily dose of 230 mg) and/or primidone (mean daily dose of 500 mg), but only experienced suboptimal tremor relief. Sixty-one percent of participants (19/31) were naïve to tremor medications at enrollment. On week 12, two participants withdrew due to pregnancy and unrelated health issues. By week 18, two more individuals withdrew due to suboptimal perceived benefit with mild arm weakness and health issues unrelated to the study. At week 24, four more participants withdrew due to other health issues, transportation concerns, insufficient perceived benefit, and one due to worsening of tremor with some hand weakness.

Seventy-seven percent of participants in this trial had whole arm tremor involving all three joints (24/31). Participants were injected in their motor dominant arm (28 right-handed, 3 left-handed) with a mean total dose of 136 ± 54 U (10.4 ± 2.3 muscles), while their non-dominant arm received 125 ± 62 U (9.8 ± 2.7 muscles) at the first treatment. By the third injection cycle, the mean dosage was optimized to 146 ± 57U (11.0 ± 1.7 muscles) and 145 ± 72 U (10.4 ± 2.6 muscles) in the motor and non-motor dominant arms, respectively (Table 2). During dose optimization for the second and third injection in the motor dominant arm, 45% (13/29), 24% (6/29), and 31% (9/29) of participants required an increase, decrease or no change in the final optimized dose, respectively. In the non-dominant arm, 59% (17/29), 17% (5/29), and 24% (7/29) of participants required an increase, decrease or no change in total arm dose, respectively. The total dosages per joint are presented (Table 3).

### 2.2. Clinical and Kinematic Efficacy Results

**Figure 1:**
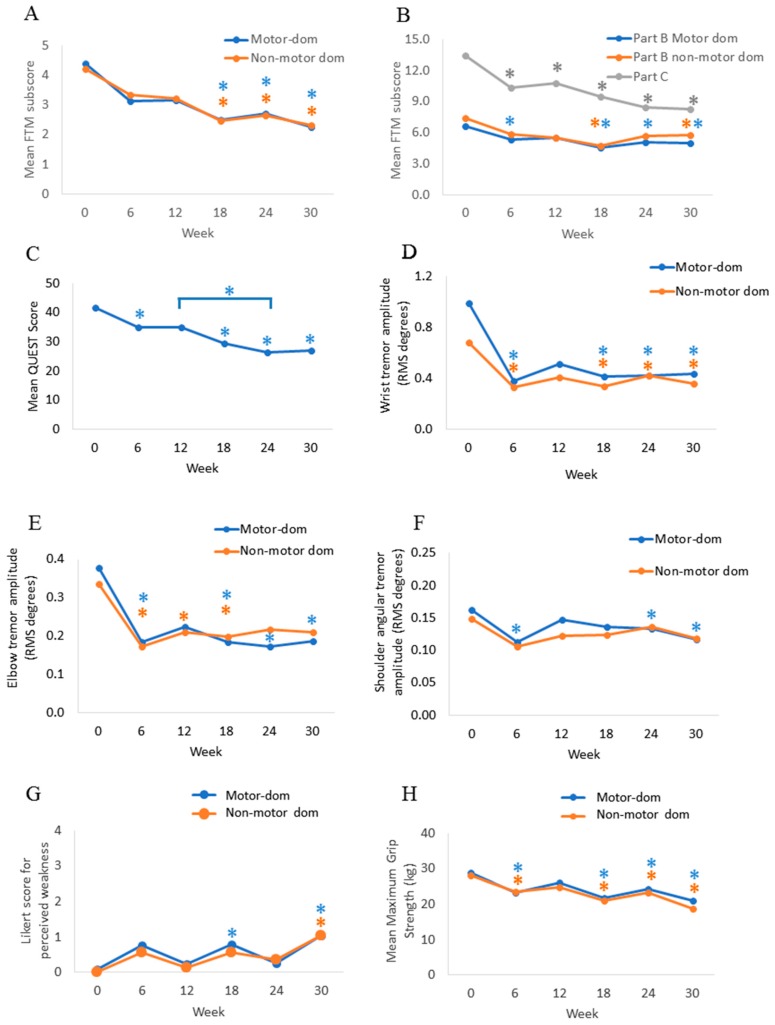
Significant reduction in tremor severity was measured clinically and objectively, and improvements in arm functionality and quality of life were observed over the 30-week treatment course. Mean clinical scores for the Fahn-Tolosa-Marin part A (**A**), part B and C (**B**), Quality of Life (**C**) scales, mean angular root mean square tremor amplitudes at the wrist (**D**), elbow (**E**), and shoulder (**F**), perceived weakness Likert scale (**G**), and mean maximal grip strength (**H**) in both upper limbs. “Motor-dom” indicates the motor-dominant arm and “non-motor dom” indicates non-motor dominant arm of all participants. Asterisks (*) represent statistical significance in means compared to week 0, or comparisons otherwise stated by a line, and asterisk colors are coordinated with each line plot. Injections were administered every 12 weeks starting at week 0.

In Figure 1a, the mean FTM part A (tremor severity) score in both limbs was significantly reduced by 42.1% (X^2^(5) = 38.743; *p* < 0.005) from 4.3 ± 1.8 FTM points at week 0 (median = 3.5) to 2.5 ± 1.8 points (median = 2.25, *p* < 0.005) at week 18 and was maintained by a 47% tremor reduction at week 30 (median = 2.0, *p* < 0.005). Fine motor skills for writing, drawing, and pouring liquids (FTM part B) were significantly improved from 6.6 ± 2.2 points (median 7.0, X^2^(5) = 32.979; *p* < 0.005) at week 0 to 5.6 ± 2.4 points (median = 5.0, *p* = 0.004) at week six in the motor dominant arm (Figure 1b). Fine motor skills significantly improved in both arms following the second and third injections at week 18 (median = 5.0, *p* < 0.005) and at week 30 (median = 5.5, *p* = 0.018), respectively. Functional impairment caused by tremor (FTM part C) was significantly reduced from 13.5 ± 2.7 points (median = 13.0, X^2^(5) = 42.116; *p* < 0.005) at week 0 to 10.3 ± 2.7 points (median = 10.0, *p* = 0.001) at week six and was further improved by 39.1% (8.2 ± 3.3 points; median = 8.0, *p* < 0.005) at week 30 (Figure 1b). 

QoL (QUEST) was significantly improved over the treatment course following the first injection (Figure 1c). A significant reduction in mean QUEST score by 16.0% (X^2^(5) = 43.112; *p* < 0.005) from 41.6 ± 14.0 points (median = 41.0) at week 0 to 35.0 ± 12.9 points (median = 36.0, *p* = 0.036) at week six was reported, and it was further improved to 27.2 ± 10.1 points (median = 30.0, *p* < 0.005) at week 30. 

Mean angular tremor amplitude across all four tasks for all participants in the motor dominant arm was significantly reduced by 61.7% (X^2^(5) = 32.195; *p* < 0.005) at the wrist, by 47.5% at the elbow (X^2^(5) = 26.386; *p* < 0.005), and by 30.0% at the shoulder (X^2^(5) = 24.054; *p* < 0.005) from week six through to week 30 (Figure 1d–f). Similarly, wrist tremor amplitude was reduced by 52.5% (X^2^(5) = 25.710; *p* < 0.005) in the non-motor dominant arm from 0.7 ± 0.9 root mean squared (RMS) degrees (median = 0.4) at week 0 to 0.3 ± 0.4 at week six (median = 0.2, *p* = 0.014) and was maintained with a 49.3% tremor amplitude reduction (0.4 ± 0.6, median = 0.2, *p* = 0.008) up to week 30. Mean elbow tremor amplitude in the non-task dominant arm was significantly reduced by 46.9% (X^2^(5) = 22.601; *p* < 0.005) from 0.3 ± 0.5 RMS degrees (median = 0.1) at week 0 to 0.2 ± 0.2 (median = 0.1, *p* = 0.003) at week six and was maintained with a 47.5% reduction (0.2 ± 0.2, median = 0.1, *p* = 0.011) up to week 18. No significant reductions in mean shoulder tremor amplitudes in the non-motor dominant arm were observed over the treatment course, however, a trend towards tremor reduction was observed. 

### 2.3. Tolerability of BoNT-A Therapy

Manual muscle testing (MMT) for finger extensor weakness (≤3 MMT rating) was assessed over 30 weeks. Out of the 136 total follow-up assessments, 17.6% (24/136) of assessments had finger weakness in either hand at any given time. Mild weakness (≤3 MMT rating) was observed at the elbow in only one participant, and no shoulder weakness was reported in any participant. The mean Likert score rated by participants for perceived muscle weakness was 0.8 ± 0.9 at week 18 (median = 1.0, *p* = 0.013) and 1.0 ± 1.2 at week 30 (median = 1.0, *p* = 0.004) in the motor dominant arm, when compared to week 0 (no weakness, median = 0.0), indicating mild yet non-bothersome hand weakness following the second and third injections (Figure 1g). The mean Likert score was 0.6 ± 0.8 at week 18 (median = 1.0, *p* = 0.012) in the non-motor dominant arm. The reduction in maximum grip strength was statistically significant (X^2^(5) = 55.755; *p* < 0.005) from 28.4 ± 10.6 kg force (median = 28.3) at week 0 to 23.4 ± 10.2 (median = 24.5, *p* = 0.001) at week six and returned to baseline strength at week 12 (Figure 1h). Mean maximal grip strength was again significantly reduced to 21.3 ± 10.2 (median = 21.2, *p* < 0.0005) at week 18, 23.8 ± 10.1 (median = 22.7, *p* = 0.003) at week 24, and 19.8 ± 9.1 (median = 18.2, *p* < 0.0005) at week 30. 

## 3. Discussion

This is the first study to demonstrate bilateral upper limb tremor treatment using BoNT-A with the successful return of arm functionality and immediate improvement in the quality of life six-weeks following the first injection cycle and was maintained up to week 30. In contrast to our past unilateral treatment study, where the most disabling arm, as perceived by the patient, was treated, in the present study, QoL was significantly improved by treating both upper limbs, resulting in a 26-week earlier improvement in QoL [3]. Additionally, QoL continued to further improve after the second and third cycles of bilateral arm therapy. This level of QoL improvement did not occur in the unilateral injection study until after the 4th injection cycle [4]. Treatment in both the motor dominant and non-motor dominant arms likely contributed to the earlier participant reported improvements. This is likely because the non-motor dominant arm is involved in numerous bimanual daily tasks, implying that providing treatment for a single limb may not be enough to improve overall task function [5,8]. Other outcomes, such as FTM part A (tremor severity), B (fine motor control), and C (functional interference by tremor for activities during daily living (ADLs)), showed significant improvements all within the first 18 weeks of treatment. 

Similar to our previous studies, maximal grip weakness did not substantially impact hand function. Participant-based assessments using the Likert scale confirmed that any perceived weakness did not impair any hand function. In all, only three participants withdrew due to the sub-optimal tremor relief and/or bothersome weakness. Current thinking has suggested that focusing toxin injections to the flexor compartment and avoiding extensor carpi muscles of the forearm is required to minimize weakness [7,9]. However, these muscles were not excluded in our study and yet wrist weakness was minimal. In addition, when all joints were injected, substantial weakness was not observed proximally in the arm. Our results suggest that there is no need to restrict injections in any muscle group and that optimal individualization is possible. Fixed-dose paradigms that are not titrated to the multi-joint and individual tremor severity cannot achieve these results [10,11].

This study used an automated, computer-assisted algorithm for the determination of injected muscles and the respective doses of BoNT-A. This is the first time that BoNT-A injection parameters have been solely selected without clinical assessment. Objective sensor-based measurement of tremor across multiple joints, subsequently analyzed by the algorithm resulted in a standardized approach to BoNT-A treatment for tremor yet providing individualized dosing. The application of such a methodology is novel in the field of BoNT-A injections. It is likely that by the computer-assisted tremor analysis, individualization and optimization of injection parameters across multiple joints ensured efficacy and a low incidence of hand weakness. 

Other research teams have now also emphasized the importance of customizing BoNT-A therapy for limb tremor [6,12], in order to minimize hand weakness. However, functional improvement in ADLs and QoL following BoNT-A therapy has only been studied and reported with kinematic-based muscle and dose selection thus far. Furthermore, the use of drug delivery devices, like needle electromyography (EMG), for probing all the muscles in the patient’s arm is discomforting, can be resource intensive in terms of expertise and time, and impractical. The EMG signal varies with the depth of the EMG needle and the angle of insertion, and thus EMG use to detect tremulous muscles is not a standardized practice. Kinematic assessment and analysis can be completed within 10–20 minutes per arm and provides injectors with data-driven clinical support for optimizing BoNT-A therapy. Our computer-assisted kinematic analysis system does not use accelerometers as they do not provide the fidelity or accuracy for multi-joint tremor characterization and breakdown for all degrees of freedom. The sensors used in this study meet this important requirement. Finally, tremor change can be graphically displayed easily in this software to manage patient expectations following treatment. 

A study limitation of our previously published tremor-toxin studies allowed the injector to adjust dosing parameters [3,4,13]. In this study, a blinded-injector approach was utilized as initial BoNT-A injection parameters were entirely based on our computer-assisted, kinematic tremor analysis, and optimization of BoNT-A injections was conducted using kinematics and any presence of weakness perceived by each participant. There was no placebo-controlled treatment group; however, three injection cycles were conducted limiting a placebo-response in participants. The study was restricted to a single-injector and the muscle selection and dosing algorithm was based on data analysis from the previous tremor study of the same injector. For future study designs, multiple injectors with a placebo-controlled arm should be considered, along with an improved dosing algorithm based on tremor outcomes from multiple injectors. 

This study shows that personalizing BoNT-A injection parameters solely from each participant’s kinematic tremor characteristics in both functionally disabled upper limbs was efficacious in maintaining tremor reduction. The ability to perform bilateral, fine, and gross motor tasks for daily and social activities significantly improved QoL over the three serial treatments. Using computer-assisted tremor analysis and treating both functionally disabling upper limbs, this study suggests that ET patients can achieve improved QoL faster while personalizing tremor treatment per arm with ease. 

## 4. Materials and Methods 

This open-label single center, single injector trial was approved by the Western University Health Sciences Research Ethics Board (REB#104584) on January 9, 2014, and was registered on the clinicaltrials.gov registry (ClinicalTrials.gov Identifier: NCT02551848) and with Health Canada (CTA#184947). First participant’s first visit and last participant’s last visit (week 30) occurred in September 2014 and April 2018, respectively. The ethics committee provided full board approval for this clinical trial protocol, and written consent was obtained from all participants. The study design and analysis is illustrated in a CONSORT flowchart (Figure S1).

A convenience sampling of 35 ET participants with functionally debilitating bilateral arm tremor was recruited from the London Movement Disorders Centre in Canada. Participants completed six study visits at weeks 0, 6, 12, 18, 24, and 30, and were treated over three serial injections with BoNT-A (incobotulinumtoxinA; Xeomin^®^) in both upper limbs at weeks 0, 12, and 24. Follow-up visits measuring peak effect of BoNT-A occurred six-weeks post-injection. Clinical scales and kinematic tremor assessment were captured at every study visit. A clinical decision on the selection of muscles to treat and dosages were initially determined based on computer-assisted analysis of tremor. BoNT-A was injected using needle electromyographic (EMG) guidance (1” long 30 g injectable EMG needle using a Myoguide^®^ portable EMG machine, Bolton, ON, Canada). 

### 4.1. Eligibility Criteria

Inclusion criteria were: female and male participants diagnosed with ET based on the Tremor Investigation Group (TRIG) criteria and had functionally disabling tremor in both upper limbs defined by the participant, either received stable but sub-optimal tremor oral medication for at least 3 months prior to enrollment or chose to skip oral medication. All individuals were BoNT-A naïve for tremor management. ET participants were required to have a disabling tremor in both their left and right arms. If any participant had a bilateral tremor but only perceived a debilitating tremor in one arm, such participants were not recruited. Exclusion criteria were: history of stroke, contradictions to BoNT-A drug monograph, pregnancy, bilateral tremor with asymmetric upper limb functional impairment, limb tremor severity that was deemed insufficient to require at least 20 U, existing pharmacological therapy with tremor-inducing side effects (e.g., lithium, valproate, steroids, amiodarone, beta-adrenergic agonists like salbutamol), and other clinically significant health issue changes that may impact study participation. No medications were withheld or adjusted during the study period.

### 4.2. Clinical Endpoints

At all visits, tremor severity and arm functionality were reported using the Fahn-Tolosa-Marin (FTM) tremor rating scale. FTM scale assessed tremor severity (part A) during posture and action were scored separately for each upper limb, ability to draw and pour liquids (part B) was scored per limb, and functional disability that impact activities of daily living caused by tremor (part C) was scored, most of which correspond with bimanual tasks [14]. QoL was reported using the quality of life for essential tremor questionnaire (QUEST), which includes 30-items assessing psychosocial, communication, and physical activities [15]. Tolerability of BoNT-A was assessed by monitoring maximal grip strength by using a Baseline^®^ hydraulic hand dynamometer (Item#:12–0240, White Plains, NY). Manual muscle testing (MMT) was used to assess the finger, wrist, and elbow flexor/extensor muscles [16,17]. A Likert participant-ranked scale to assess participant perceived muscle weakness was used (ranging from 0: no weakness, 1: mild but not bothersome, 2: moderate, 3: marked, and 4: severe weakness with functional loss in injected muscles). 

### 4.3. Kinematic Procedure

Upper limb kinematics was captured using three goniometers and a torsiometer placed over the forearm, wrist, elbow, and shoulder joints. Participants performed four scripted tasks for measuring tremor at each joint: two postural positions with arms pronated outstretched with palms facing the ground (“posture-1”) or outstretched arms semi-supinated with palms facing the other (“posture-2”), and two weight-bearing tasks where participants held an empty cup (“load-1”) or held a cup with an additional 1-lb weight inside the cup (“load-2”). Raw data was recorded by the Biometrics DataLINK acquisition system (PC Software version 8.7) at a sampling rate of 200 Hz. The computer-algorithm was written in MatLab^®^ (Version 2014b) software. The tremor analysis provided each participant’s tremor characteristics: amplitude (angular root mean squared (RMS) degrees) at the wrist, elbow, and shoulder joints for each scripted task [4]. Furthermore, wrist and shoulder tremors were separated into the distribution of muscle groups for each task: wrist flexion-extension, pronation-supination and radial-ulnar deviations, forearm flexion-extension, and shoulder flexion-extension and abduction-adduction.

### 4.4. BoNT-A Injection Parameter Selection Using Computer-Assisted Tremor Analysis

Participants received three cycles every 12 weeks of bilateral arm intramuscular injections of BoNT-A (total arm dose ranging from 20–300 U) into identified muscles groups that contributed to the multi-joint tremor. For all BoNT-A injections, parameters were determined by the computerized kinematic tremor analysis per participant per arm. The first dose and serial optimization of BoNT-A parameters were determined from previous studies conducted by the London Movement Disorders Centre involving 24 ET and 28 Parkinson disease (PD) participants over 3 injection cycles [3,13]. For the first injection, a dosing algorithm incorporated tremor severity, chosen injection parameters, and subsequent clinical and kinematic outcomes following the first injection cycle. Subsequent BoNT-A dose optimization was based on a change in tremor kinematic severity and any presence of arm weakness while maximizing tremor suppression.

### 4.5. Statistical Analyses

No formal sample size calculation was performed, and all analysis was exploratory. For all participants, their right and left tremor arm were categorized as either motor dominant or non-motor dominant arm during data analysis to reflect which arm was frequently used during activities of daily living (ADL). Mean tremor RMS amplitudes and clinical rating scores were plotted for all time-points. IBM^®^ SPSS^®^ statistics version 20 was used to analyze kinematic and clinical data using a non-parametric Friedman one-way repeated measures analysis of variance (ANOVA) test using confidence intervals of 95% (α = 0.05) with post-hoc Bonferroni corrections for multiple comparisons performed from baseline (week 0) to all time-points (weeks 6 to 30), at peak effect of BoNT-A (weeks 6, 18, 30), and at re-injection visits (weeks 0, 12, 24).

## 5. Patents

Two patents, PCT/CA2013/000804 and PCT/CA2014/050893 pending to MDDT Inc. (London, ON, Canada), resulted from the work reported in this manuscript.

## Figures and Tables

**Table 1:** Participant demographics and baseline quality of life (QUEST), tremor severity and arm functionality (FTM parts A-C) scores for all participants.

Patient	Gender	Age	Tremor Medication List (Total Daily Dose)	Motor-Dominant Hand	Baseline Scores					
						Motor-Dominant Limb		Non Motor-Dominant Limb		
					QUEST Score (/120)	Total FTM Part A (/12)	Total FTM Part B (/16)	Total FTM Part A (/12)	Total FTM Part B (/16)	Total FTM Part C (/32)
1	M	76	N/A	R	37	1	7	2	6	15
2	M	63	N/A	R	69	6	10	9	12	14
3	M	64	Primidone (750 mg)	R	73	6	7	4	4	22
4	F	76	Primidone (500 mg), Propranolol (250 mg)	R	44	5	6	6	9	17
5	M	72	N/A	L	24	3	5	4	4	13
6	F	69	Primidone (500 mg), Propranolol (180 mg)	R	30	6	8	9	12	14
7	M	70	N/A	R	49	6	8	6	6	15
8	F	65	N/A	R	39	5	2	2	1	9
9	F	79	Propranolol (120 mg)	R	21	2	7	2	8	12
10	M	67	Propranolol (500 mg)	R	45	6	10	8	12	12
11	F	66	Propranolol (250 mg)	L	44	3	6	5	5	13
12	M	68	N/A	R	33	4	6	4	5	12
13	F	52	N/A	R	56	3	4	5	5	10
14	F	61	Gabapentin (900 mg)	R	56	3	8	3	7	13
15	F	77	N/A	R	31	3	7	3	6	12
16	F	75	N/A	R	33	4	5	2	2	9
17	M	75	N/A	R	26	4	7	3	7	12
18	F	63	Gabapentin (900 mg)	R	71	4	9	3	10	16
19	M	78	N/A	R	32	4	8	3	7	14
20	M	69	N/A	R	50	2	6	3	4	12
21	M	71	Primidone (250 mg)	R	31	5	5	6	5	11
22	M	77	N/A	R	17	4	4	5	9	11
23	F	65	Primidone (500 mg)	R	53	4	5	5	15	19
24	M	74	Propranolol (80 mg)	R	37	4	4	3	5	14
25	M	83	N/A	R	50	6	8	1	6	16
26	M	70	N/A	R	44	8	7	3	4	15
27	M	71	N/A	R	32	4	8	7	14	13
28	M	70	N/A	L	41	4	15	5	13	15
29	M	65	Topiramate (100 mg)	R	50	4	7	5	11	14
30	F	65	N/A	R	42	4	6	3	4	13
31	M	64	N/A	R	31	5	5	6	8	11
Mean	12F	69.7	19 received BoNT-A monotherapy	3L	41.6	4.3	6.8	4.4	7.3	13.5
SD		6.5			14.0	1.5	2.4	2.1	3.6	2.7
Range		52–83			17–73	1–8	2–8	1–9	1–15	9–22

Medications listed represent current, concomitant treatment at the time of BoNT-A therapy. Abbreviations: FTM: Fahn-Tolosa-Marin tremor rating scale; QUEST: quality of life for essential tremor questionnaire; SD: standard deviation.

**Table 2:** Total BoNT-A dosages injected per upper limb for each participant over the three injection treatments.

Patient	Total Arm Dose					
	Week 0 (1st Injection) (N = 31)		Week 12 (2nd Injection) (N = 29)		Week 24 (3rd Injection) (N = 23)	
	Motor Dominant	Non-Motor Dominant	Motor Dominant	Non-Motor Dominant	Motor Dominant	Non-Motor Dominant
1	160	100	225	160	270	205
2	190	160	190	225	190	235
3	190	120	180	110	No injection (a)	
4	175	215	170	150	No injection (c)	
5	100	119	105	140	No injection (c)	
6	270	300	195	215	185	205
7	160	105	No injection (a)			
8	90	60	155	105	150	70
9	60	60	60	60	60	60
10	180	260	180	300	180	300
11	100	110	100	110	100	110
12	100	100	130	135	130	135
13	70	110	No injection (b)			
14	200	125	200	125	185	125
15	195	155	195	155	195	155
16	100	55	100	55	100	55
17	140	110	195	110	205	140
18	100	145	125	180	135	210
19	100	120	200	210	230	240
20	120	175	120	175	130	185
21	120	100	145	100	145	100
22	150	120	100	155	No injection (a)	
23	120	175	165	240	160	235
24	155	140	155	170	No injection (c)	
25	145	30	155	30	155	45
26	180	100	210	110	No injection (d)	
27	100	120	100	145	100	135
28	115	115	140	140	90	90
29	240	200	165	165	165	165
30	35	20	35	20	35	20
31	60	40	65	85	65	110
Mean	136.1	124.6	146.9	140.7	146.1	144.8
SD	54.1	61.8	48.9	62.2	57.0	73.1
Range	20–100	20–120	30–90	30–80	40–100	35–100

^a^ Other health concerns ^b^ Pregnancy ^c^ Limited perceived benefit and/or arm muscle weakness ^d^ Travel concerns.

**Table 3:** Mean BoNT-A dosages injected into the wrist, elbow, and shoulder muscle groups for all participants over the three injection treatments.

	Total Dose Per Limb Joint					
	Week 0 (1st Injection) (N = 31)		Week 12 (2nd Injection) (N = 29)		Week 24 (3rd Injection) (N = 23)	
	Motor Dominant	Non-Motor Dominant	Motor Dominant	Non-Motor Dominant	Motor Dominant	Non-Motor Dominant
Wrist Mean	50.2	46.0	54.0	54.6	53.0	53.7
SD	22.1	22.3	20.3	22.3	25.6	26.2
Range	20–100	20–120	30–100	20–110	30–130	15–110
Elbow Mean	44.1	42.8	46.8	46.1	47.5	50.5
SD	17.0	18.7	15.6	17.7	14.6	21.3
Range	0–80	0–80	0–90	0–90	0–80	0–90
Shoulder Mean	51.3	49.8	55.4	54.8	54.8	60.3
SD	15.7	16.9	18.5	18.9	20.0	23.3
Range	0–100	0–100	0–100	0–100	0–100	0–100

## Supplementary Materials

The following are available online at http://www.mdpi.com/2072-6651/11/2/125/s1, Figure S1: Study design, follow-up, and analysis of participant datasets in the form of a CONSORT flow diagram.
