# Supplementary figures and images for: Personalized Bilateral Upper Limb Essential Tremor Therapy with Botulinum Toxin Using Kinematics
Source: Toxins (Basel). 2019 Feb 19;11(2):125. doi: 10.3390/toxins11020125 (PMC6409675; doi:10.3390/toxins11020125)

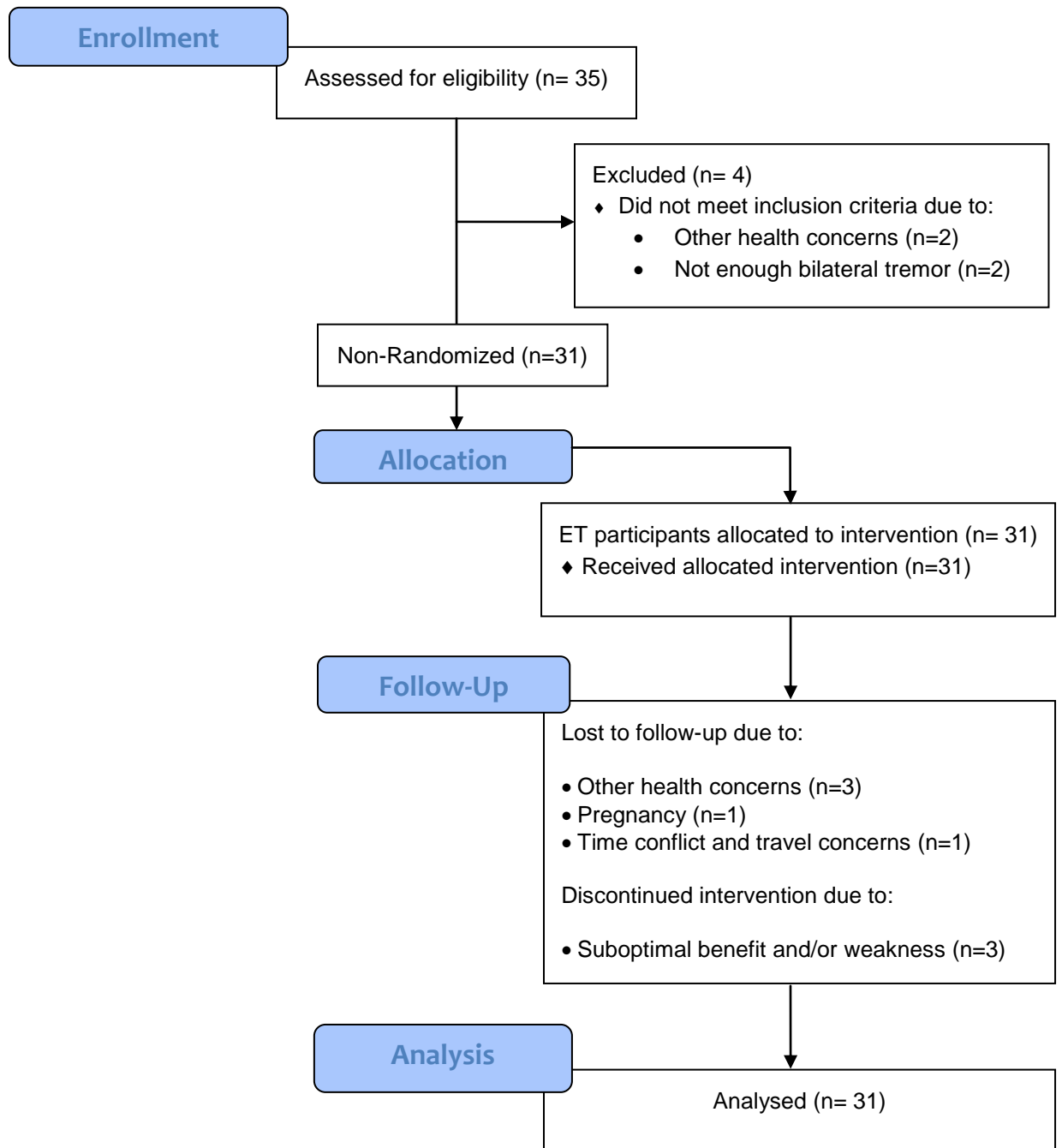

Supplement: Supplementary file 1 [file toxins-11-00125-s001.zip › toxins-448585 SPM.pdf]
